# A Polyclonal Immune Function Assay Allows Dose-Dependent Characterization of Immunosuppressive Drug Effects but Has Limited Clinical Utility for Predicting Infection on an Individual Basis

**DOI:** 10.3389/fimmu.2020.00916

**Published:** 2020-05-15

**Authors:** Stefanie Marx, Claudia Adam, Janine Mihm, Michael Weyrich, Urban Sester, Martina Sester

**Affiliations:** ^1^Department of Transplant and Infection Immunology, Saarland University, Homburg, Germany; ^2^Department of Internal Medicine IV, Saarland University, Homburg, Germany

**Keywords:** immunomonitoring, transplantation, immunosuppression, infection, pharmacodynamics, pharmacokinetics, T-cell

## Abstract

Dosage of immunosuppressive drugs after transplantation critically determines rejection and infection episodes. In this study, a global immune function assay was characterized among controls, dialysis-patients, and transplant-recipients to evaluate its utility for pharmacodynamic monitoring of immunosuppressive drugs and for predicting infections. Whole-blood samples were stimulated with anti-CD3/toll-like-receptor (TLR7/8)-agonist in the presence or absence of drugs and IFN-γ secretion was measured by ELISA. Additional stimulation-induced cytokines were characterized among T-, B-, and NK-cells using flow-cytometry. Cytokine-secretion was dominated by IFN-γ, and mainly observed in CD4, CD8, and NK-cells. Intra-assay variability was low (CV = 10.4 ± 6.2%), whereas variability over time was high, even in the absence of clinical events (CV = 65.0 ± 35.7%). Cyclosporine A, tacrolimus and steroids dose-dependently inhibited IFN-γ secretion, and reactivity was further reduced when calcineurin inhibitors were combined with steroids. Moreover, IFN-γ levels significantly differed between controls, dialysis-patients, and transplant-recipients, with lowest IFN-γ levels early after transplantation (*p* < 0.001). However, a single test had limited ability to predict infectious episodes. In conclusion, the assay may have potential for basic pharmacodynamic characterization of immunosuppressive drugs and their combinations, and for assessing loss of global immunocompetence after transplantation, but its application to guide drug-dosing and to predict infectious on an individual basis is limited.

## Introduction

Immunosuppressive drug treatment and the development of new drugs have made significant contributions to improved outcome and survival of transplanted organs over the last decades. The net state of immunosuppression in transplant-recipients is interindividually variable and may be influenced by the dosage and combination of drugs, but also by age and other comorbidities. The choice of immunosuppressive drugs and their dosage influences clinical outcome in that underimmunosuppression favors rejection episodes and overimmunosuppression is associated with an increased incidence of infectious complications. This is particularly evident in the first months after transplantation, when immunosuppression is highest. As not all patients develop clinical complications, assays for monitoring of drug effects and for predicting infectious complications are needed for personalized management of immunosuppressive and antimicrobial drug therapy.

At present, immunosuppressive drugs are either prescribed in empirically defined dosages or based on determination of individual drug-levels from blood. As this pharmacokinetic type of drug-dosing does not take interindividual differences in drug-susceptibility into account, pharmacodynamic monitoring of immunosuppressive drug effects on major lymphocyte populations has been explored as a promising approach toward individualization of immunosuppressive drug treatment (1). Pharmacodynamic principles include assessment of the inhibitory action of drugs on the activity of enzymatic drug-targets or on transcription factors, on ATP function or on cytokine induction (2, 3). Disadvantages of these approaches include requirement of isolated blood cells and use of aqueous media under non-physiological conditions (3, 4). In this regard, whole blood assays may be favorable, as little sample manipulation is involved, and immunosuppressive drugs are present in clinically relevant concentrations and therefore closely represent physiological conditions *in vivo*. This is illustrated in whole blood samples stimulated with viral or bacterial antigens, where calcineurin-inhibitors exert a dose-dependent inhibitory action on intracellular cytokine induction in T-cells (5–7). Based on the choice of stimuli, these analyses were restricted to T-cells, and did not provide any information on the inhibitory action of immunosuppressive drugs on lymphocyte subpopulations of the innate immune system. This may be of interest, as NK-cell or Toll-like receptor (TLR) polymorphisms or altered complement levels were shown to be associated with an increased incidence of infections (8–10).

The QuantiFERON monitor assay (Qiagen, Hilden, Germany) is a novel global immune assay that is based on induction of IFN-γ from whole blood samples after stimulation with a lyosphere consisting of a T-cell stimulus (anti-CD3) and a TLR7/8-agonist (R848). It therefore targets cellular components of both the adaptive and the innate immune response, although the relative contribution of these subpopulations toward IFN-γ production is currently unknown. The assay may hold promise as a tool to predict infections after transplantation (11, 12). Although IFN-γ secretion-levels were shown to be lower in transplant-recipients as compared to controls (11–15), the utility of this assay for pharmacodynamic analysis of immunosuppressive drugs is less well-characterized.

This study was carried out to characterize the cytokine-secreting cellular subpopulations and their dominant cytokine patterns in response to stimulation with the lyosphere. Moreover, the assay was used to evaluate its utility for pharmacodynamic analysis of the inhibitory effect of immunosuppressive drugs and drug-combinations *in vitro* and *in vivo* in clinically relevant dosages. Finally, immune function of transplant-recipients was analyzed before and during the first year after transplantation to assess its utility to predict infectious complications.

## Materials and Methods

### Subjects

Immunocompetent healthy controls were recruited to characterize cell populations and cytokines after stimulation with the QuantiFERON monitor assay, to study the inhibitory effect of immunosuppressive drug and drug-combinations *in vitro*, and to study natural diurnal variations in immune-reactivity, which included whole blood samples collected at five times over 24 h. To analyze the effect of various levels of immunodeficiency on cellular immune function, immunocompetent controls, dialysis patients, short-term and long-term transplant-patients (transplantation <3 months and more than 1 year ago, respectively) were recruited. Moreover, renal transplant-patients were prospectively and consecutively enrolled and immune function was analyzed before and 1, 3, 6, 12 months after transplantation and associated with infectious episodes. The study was approved by the local ethic committee (126/14, Ärztekammer des Saarlandes), and all individuals gave written informed consent.

### Clinical Outcomes

The occurrence of infections was recorded during the first year after transplantation based on definitions for use in clinical trials involving organ transplant-recipients (16). The records of all patients were screened for bacterial, viral and fungal infections. Infectious episodes were defined microbiologically or based on clinical diagnosis and/or response to treatment.

### Quantification of Lymphocyte Subpopulation and Polyclonal Cell Reactivity

Lymphocytes subsets were identified using cell surface staining of CD3, CD4, CD8 (T-cells), CD16/CD56 (NK-cells), and CD19 (B-cells) from whole blood after staining with fluorescently labeled antibodies (anti-CD3, anti-CD4, anti-CD8, anti-CD16/56, anti-CD19, all from BD) for 30 min at room temperature as described before (7, 17). Samples were analyzed on a FACS Canto II using DIVA Software (BD).

### Stimulation and Quantitation of Polyclonal Cell Reactivity

Heparinized blood samples were polyclonally stimulated using the QuantiFERON monitor assay (a lyopsphere containing anti-CD3 antibodies and the TLR7/8 agonist R848) for 20 h at 37°C at 5% CO_2_ according to the manufacturer's instructions (Qiagen, Hilden, Germany). Thereafter, cell-free supernatants were frozen at −20°C, and IFN-γ in the supernatant was quantified by ELISA. Replicates or longitudinal samples of one individual were analyzed together on one ELISA plate. In addition, blood samples were stimulated with the lyosphere or with 2.5 μg/ml *Staphylococcus aureus* enterotoxin B (SEB) for intracellular cytokine staining. Samples were stimulated for 16 h at 37°C, before adding 10 μg/ml brefeldin A. Four hours later, cells were treated with 2 mM EDTA for 15 min. Thereafter, samples were treated with lysing solution (BD). Fixed cells were washed with FACS buffer (PBS-5%FCS-0.5%BSA-0.07%NaN_3_) and subsequently treated with 0.1% saponin for 10 min. The surface markers for T-, B-, and NK-cells (CD3, CD4, CD8, CD19, CD16/56), CD69 as activation marker, and cytokines (IFN-γ, IL-2, TNF-α, IL-4, IL-17) were stained with fluorescent antibodies (all from BD) and analyzed by flow-cytometry.

### Quantification of the Immunosuppressive Effect of Calcineurin Inhibitors and Steroids *in vitro*

Heparinized blood from 10 immunocompetent individuals was preincubated for 2 h with increasing doses of methylprednisolone (10, 100, 1,000 ng/ml), cyclosporine A (0, 41, 123.4, 370.3, 1111, 3333 ng/ml), and tacrolimus (0, 2.47, 7.4, 22.2, 66.6, 200 ng/ml) individually and in combination (370.3 ng/ml cyclosporine A+100 ng/ml methylprednisolone; 22.2 ng/ml tacrolimus+100 ng/ml methylprednisolone). Thereafter, cells were stimulated with the lyosphere and IFN-γ secretion was analyzed by ELISA.

### Determination of Endogenous Cortisol Levels

Cortisol levels were determined from plasma samples by the central laboratory at Saarland University Medical center using a solid-phase radioimmunoassay (Ciba Corning, Berlin, Germany).

### Statistical Analyses

Statistical analyses were performed with the GraphPad Prism Version 5.03. Nonparametric analyses from two or more groups of non-paired samples were performed using the Mann-Whitney or Kruskall-Wallis-test, analyses from paired samples were done using the Friedman test with Dunn's *post-test*. The repeated measures ANOVA with Tukey's post-test was performed for paired analyses of parametric values. Correlations were analyzed according to Spearman.

## Results

### CD4 T-Cells, CD8 T-Cells, and NK-Cells as Main Populations Producing Interferon-γ After Polyclonal Stimulation With Anti-CD3 and a TLR7/8 Agonist

**Figure 1:**
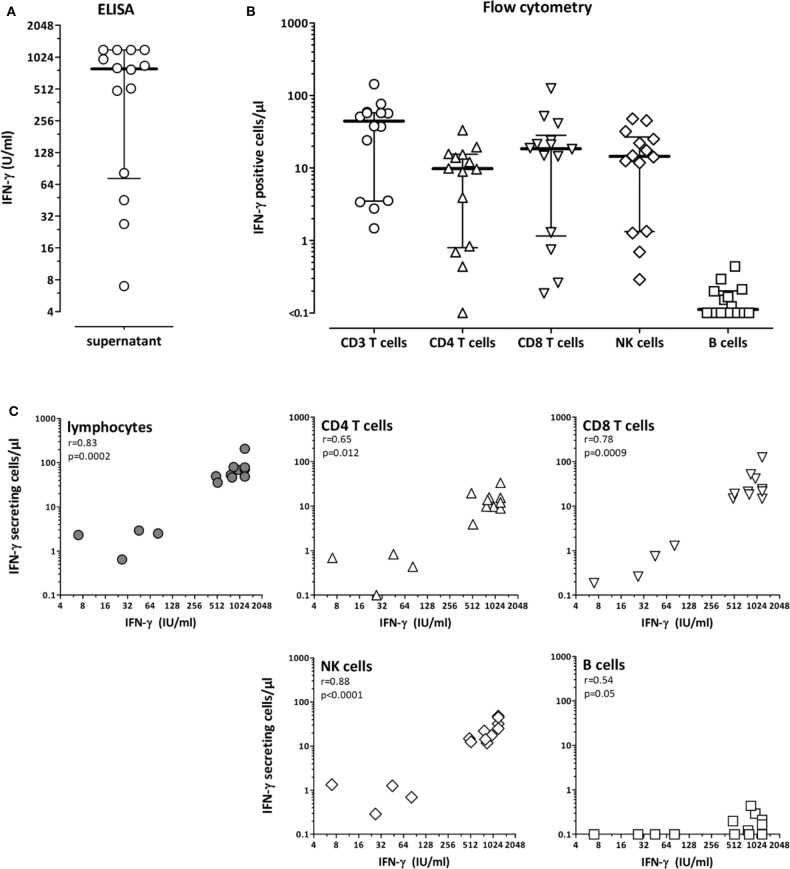
CD4 T-cells, CD8 T-cells and NK-cells as main populations producing interferon-γ after polyclonal stimulation with the lyosphere. Whole blood samples from 14 healthy controls (31.8 ± 7.7 years of age, 10 females) were stimulated for 20 h with the lyosphere and **(A)** interferon-γ (IFN-γ) was analyzed from the supernatant. **(B)** In parallel, IFN-γ producing cell populations were identified and quantified after stimulation and intracellular cytokine staining. Absolute numbers of IFN-γ producing cells were based on flow-cytometric analyses and differential blood counts. **(C)** The number of IFN-γ producing lymphocytes and lymphocyte subpopulations was correlated with IFN-γ release into the supernatant. Correlations were calculated according to Spearman.

To determine the cells that produce IFN-γ after stimulation using the QuantiFERON monitor assay, whole blood from 14 immunocompetent individuals was stimulated for 24 h and cytokine-producing cell subpopulations were determined using flow-cytometry. IFN-γ secretion into the supernatant by ELISA was determined in parallel, which showed a large interindividual variability with median IFN-γ levels of 792.4 IU/ml (IQR 72.7-1200 IU/ml, Figure 1A). Flow-cytometric analysis identified CD4 and CD8 T-cells and NK-cells as the main populations producing IFN-γ, whereas IFN-γ expression in B-cells was negligible (Figure 1B). Accordingly, IFN-γ secretion-levels from the supernatant strongly correlated not only with the number of IFN-γ-producing lymphocytes, but also with the number of cytokine-producing CD4, CD8, and NK-cells (Figure 1C).

We also investigated other cytokines with important functions in the cellular immune response. After simultaneous staining of IFN-γ, IL-2 and TNF-α, IFN-γ was by far the most dominant cytokine produced by both CD4 and CD8 T-cells (supporting Figure S1A). Most cells produced IFN-γ only, whereas only low percentages of cells expressed TNF-α or IL-2 alone or in combination with IFN-γ. Apart from T-cells, NK-cells were also stained for expression of IFN-γ and TNF-α. As with T-cells, NK-cells predominantly produced IFN-γ only (Figure S1B). Among CD4 and CD8 T-cells, the percentage of IFN-γ expressing T-cell populations was further compared with cells expressing IL-4 or IL-17. As shown in Figure S1C, CD4 T-cells producing IFN-γ predominated, followed by Th2 and Th17 cells (*p* < 0.0001). A similar distribution of IFN-γ, IL-4 and IL-17 expressing cells were found among CD8 T-cells. Taken together, although the lyosphere induced a variety of cytokines, IFN-γ was predominantly secreted by all tested cell populations.

### Natural Diurnal Variation in IFN-γ Production

**Figure 2:**
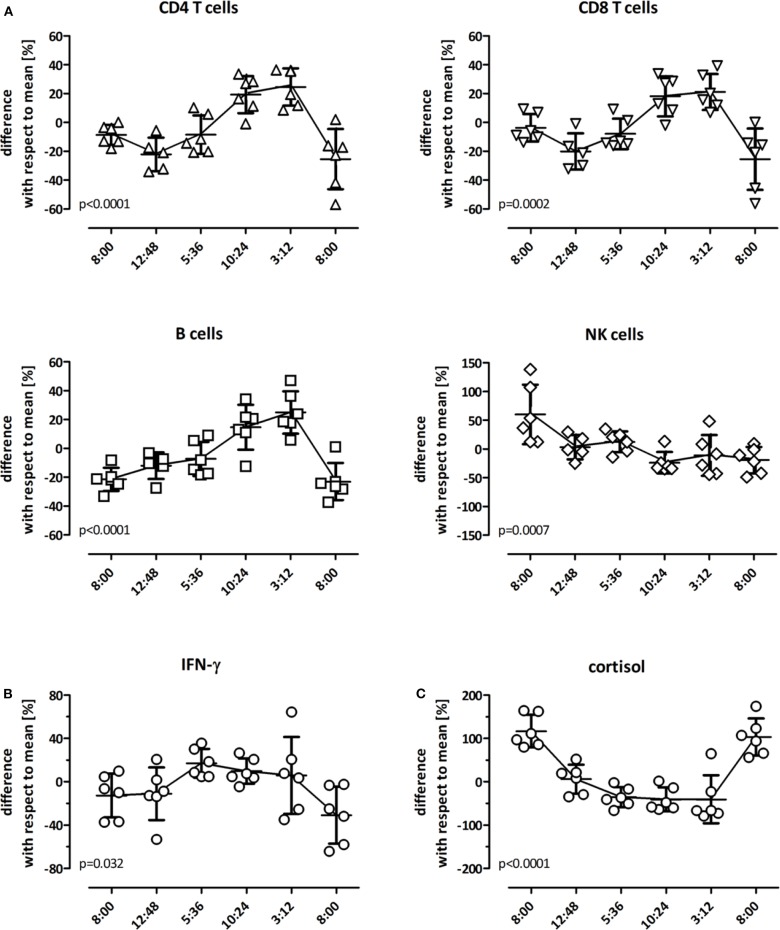
Diurnal variation in lymphocyte numbers and IFN-γ secretion. Diurnal variation of lymphocyte subpopulations in peripheral blood of healthy controls (*n* = 6) was determined over 24 h at 8:00 a.m., 12:48 p.m., 5:36 p.m., 10:24 p.m., 3:12 a.m. and the following day at 8:00 a.m. All individuals had a regular day-night rhythm. Shown are the differences in absolute cell numbers of **(A)** CD4 T-cells, CD8 T-cells, B-cells, and NK-cells and **(B)** in levels of IFN-γ and **(C)** cortisol at each time point in relation to the daily mean that was calculated from all values analyzed over the 24 h-time period (0 on the y-axis). A differential blood count to calculate absolute values was missing in one individual at 12:48 p.m. The variance at each time point with respect to this 24 h-mean is expressed as mean ± standard error of the mean. Statistical analysis was performed using the repeated measures ANOVA with Tukey's post-test.

As even immunocompetent individuals show diurnal variations in endogenous cortisol levels, potential natural variations in cell function in the absence of iatrogenic immunosuppression was studied in 6 immunocompetent individuals over a time period of 24 h. In each individual, 6 blood samples were drawn (8:00 a.m., 12:48 p.m., 5:36 p.m., 10:24 p.m., 3:12 a.m., and 08:00 a.m. at the following day), and stimulated with the lyosphere. Differential blood counts were determined in parallel. When quantifying the major lymphocyte subpopulations, diurnal dynamics of CD4 T-cells, CD8 T-cells and B-cells were similar, with cell counts being lowest during the day and highest at night (Figure 2A). In contrast, NK-cells showed different kinetics with stronger variations during the 24 h period and an overall decrease in measurable cell numbers from 1 day to the other (Figure 2A). Interestingly, IFN-γ levels were highest between evening and early morning hours (Figure 2B). This not only followed dynamics of the major lymphocyte subpopulations but also inversely correlated with endogenous cortisol levels (Figure 2C). Together this shows that IFN-γ secretion was highest during night hours and was influenced by diurnal variations in lymphocyte numbers and endogenous cortisol levels.

### Dynamics of T-Cell Functionality Over Time in the Absence of Apparent Clinical Symptoms

**Figure 3:**
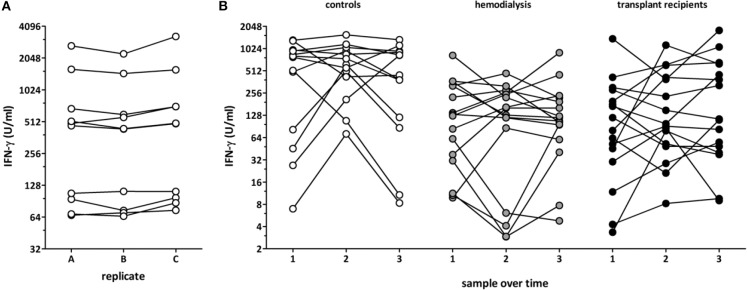
Dynamics of polyclonal T-cell functionality in asymptomatic individuals. Blood samples from healthy controls (*n* = 10; 53.4 ± 20.6 years of age, 6 females) were recruited and **(A)** three replicates of the same samples were stimulated in parallel and IFN-γ was determined from the supernatants. **(B)** Blood samples from 13 healthy controls (32.1 ± 7.9 years of age, 9 females), 16 hemodialysis patients (67.4 ± 10.9 years of age, 6 females), and 16 long-term transplant-recipients (56.4 ± 14.5 years of age, 6 females) were collected over time (3 samples per person, 35 (IQR 34) days between each time point), and stimulation-induced IFN-γ was determined from the supernatants.

To assess the intra-assay variability, three independent stimulatory reactions were performed from the same blood samples of ten healthy controls. IFN-γ levels showed a low inter-assay variability with a coefficient of variation (CV) of 10.4±6.2% (Figure 3A). To investigate the inter-assay variability, blood samples from 13 healthy individuals, 16 dialysis patients and 16 long-term transplant-patients who were apparently healthy were stimulated with the lyosphere at three different time points at a median of 35 (IQR 34) days apart from each other. Interestingly, despite absence of apparent clinical symptoms, cytokine levels determined from the supernatants within one individual showed a considerably strong variation over time in all groups with a similarly high CV of 65.0 ± 35.7% in all groups (Figure 3B, 64.1 ± 49.2% for controls, 68.8 ± 34.9% for hemodialysis patients and 62.0 ± 23.4% for long-term transplant-recipients).

### Strong Effect of Immunosuppressive Drugs on Stimulation-Induced IFN-γ Secretion *in vitro*

**Figure 4:**
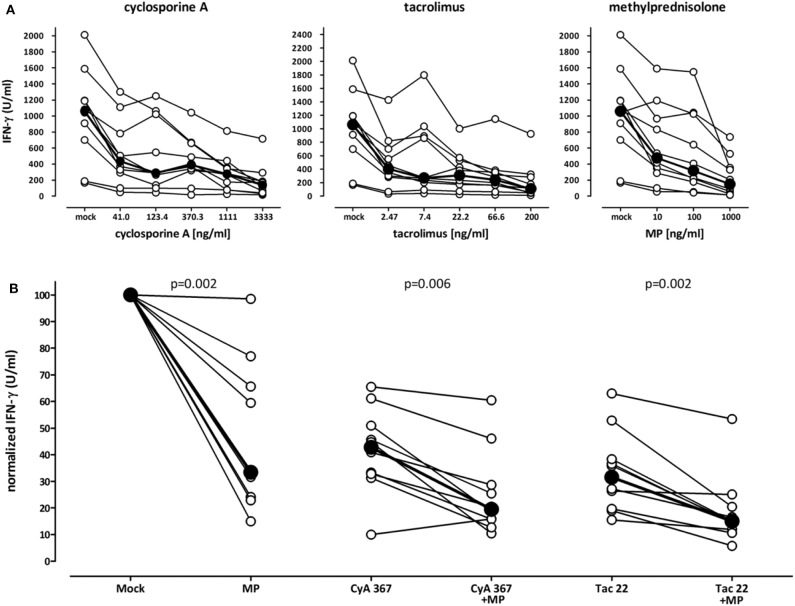
Decreasing effect of immunosuppressive drugs on stimulation-induced IFN-γ secretion *in vitro*. **(A)** Whole blood samples of ten healthy individuals (35.3 ± 12.3 years of age, 7 females) were stimulated with the lyosphere in the absence (mock) or presence of increasing dosages of cyclosporine A, tacrolimus or methylprednisolone, and IFN-γ was determined from the supernatants. Samples were pre-incubated with the drugs for 2 h prior to stimulation. Absolute levels of IFN-γ are displayed. Bold dots represent the medians connected by lines. **(B)** Blood samples were stimulated in the absence of immunosuppressive drugs (mock) or high dose methylprednisolone (MP, 1,000 ng/ml), cyclosporine A (CyA, 367 ng/ml) or tacrolimus (Tac, 22 ng/ml) as well as respective combinations. Normalized values are displayed. The level of IFN-γ in mock stimulated samples was set to 100%. Bold dots represent the medians connected by lines. Statistical analysis was performed using the Wilcoxon *t*-test. Data are expressed as median with interquartile range.

To analyze the influence of immunosuppressive drugs on cell functionality *in vitro*, blood samples from 10 immunocompetent controls were pre-treated for 2 h with increasing dosages of cyclosporine A, tacrolimus and methylprednisolone before stimulation with the lyosphere (Figure 4). As compared to samples incubated in the absence of drugs (mock), all three drugs led to a strong dose-dependent inhibition in IFN-γ secretion (Figure 4A). This immune reactivity was further reduced, when cyclosporine A or tacrolimus were combined with methylprednisolone (Figure 4B). While 367 ng/ml cyclosporine A treatment led to a decrease in IFN-γ secretion by 57.1%, the combination with methylprednisolone led to a further decrease (by 75.6%). Likewise, median secretion was reduced by 62% with 22.2 ng/ml tacrolimus and further decreased when combined with steroids (by 83.3%). Thus, both cyclosporine A and tacrolimus show a synergistic inhibitory action with steroids which can be analyzed directly from whole blood *in vitro*.

### IFN-γ Secretion Is Associated With the Level of Immunodeficiency in Patient Samples but Does Not Predict Infections on an Individual Basis

**Figure 5:**
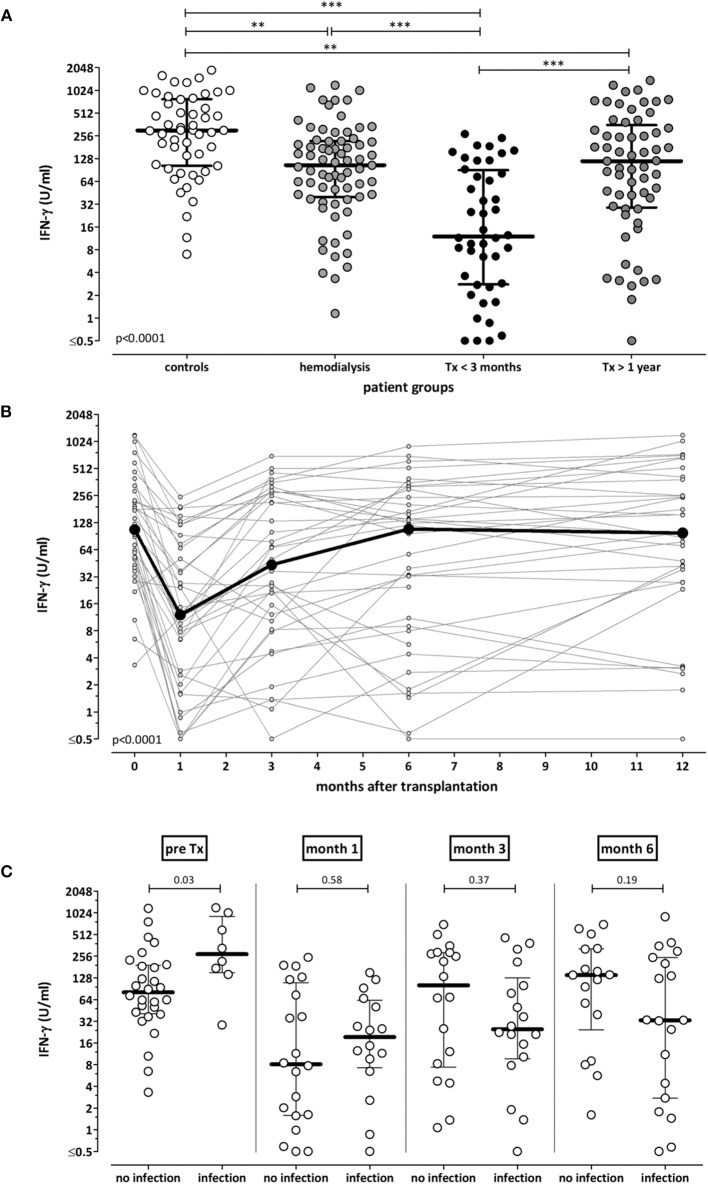
Association of IFN-γ secretion-levels with immunodeficiency and infectious episodes. **(A)** Whole blood samples of 51 healthy individuals, 71 hemodialysis patients, 44 short term and 61 long-term transplant-recipients were stimulated with the lyosphere, and IFN-γ was determined from the supernatants. Median levels of IFN-γ are indicated as bold line for each group. Statistical analysis was performed using the Kruskall Wallis test with Dunn's post-test. **(B)** Whole blood samples of 36 renal transplant-recipients were collected before, as well as 1, 3, 6, and 12 months after transplantation. Samples were stimulated with the lyosphere, and IFN-γ was determined from the supernatants. ELISA analysis was missing in two out of 36 patients. Statistical analysis was performed using the Friedman test. **(C)** Episodes of bacterial and viral infections were recorded in the 36 renal transplant-recipients throughout the first year after transplantation. IFN-γ secretion-levels before as well as 1, 3, and 6 months after transplantation were stratified according to whether patients underwent a subsequent episode of infection. Statistical analysis was performed using the Mann-Whitney test. **p < 0.01; ***p < 0.001.

To characterize test performance in patients *ex vivo*, cross-sectional analyses of samples from 51 controls and from patients with different levels of immunodeficiency was performed [71 dialysis patients (67 hemodialysis, 4 continuous ambulatory peritoneal dialysis), 44 short-term and 61 long-term transplant-patients]. The demographic and clinical characteristics are shown in Table S1. As expected, immunocompetent controls had highest secretion-levels of IFN-γ (median 303.6 IU/ml), whereas median levels were significantly lower in immunocompromized patients (Figure 5A, *p* < 0.001). Among patients, short-term transplant-patients had lowest IFN-γ secretion-levels (median 12.0 IU/ml), whereas long-term transplant-recipients reached similar median levels (118.9 IU/ml) as dialysis patients prior to transplantation (104.9 IU/ml). As most transplant recipients received tacrolimus as part of their immunosuppressive drug regimen, the immune function of these patients was analyzed in relation to tacrolimus trough levels. Short term and long-term transplant recipients differed in median tacrolimus trough levels [8.5 (IQR 7.2–10.6) ng/mL vs. 6.2 (IQR 4.7–7.3) ng/mL, *p* < 0.0001, Table S1], and corresponding levels of tacrolimus and secreted IFN-γ showed an inverse correlation (*r* = −0.23, *p* = 0.037).

In addition, IFN-γ secretion-levels of 36 renal transplant-recipients were monitored longitudinally before as well as one, three, 6 and 12 months after transplantation. Demographic and clinical characteristics as well as immunosuppressive drug dosages and tacrolimus trough levels are shown in Table 1. In line with highest levels of immunosuppressive drugs in the first weeks after transplantation, IFN-γ levels were lowest after 1 month and increased again upon tapering of immunosuppressive drugs to reach pre-transplant levels in after 12 months (*p* < 0.0001, Figure 5B). Accordingly, corresponding tacrolimus through levels were highest after 1 month and significantly lower thereafter (Table 1, *p* < 0.0001).

**Table 1:** Characteristics of renal transplant-recipients analyzed before and within the first year after transplantation.

***n (%)***		**36**			
Years of age (mean ± SD)		52.8 ± 15.5			
Females *n* (%)		12 (33.3%)			
**UNDERLYING DISEASE**					
Glomerulonephritis		17 (47.2%)			
Polycystic kidney disease		5 (13.9%)			
Vascular/hypertensive nephropathy		5 (13.9%)			
Diabetes mellitus I or II		2 (5.6%)			
Tubulointerstitial nephritis		2 (5.6%)			
Unknown/other		5 (13.9%)			
Years of renal replacement therapy (mean ± SD)		4.8 ± 3.9			
Previous transplants		First (*n* = 34)/second (*n* = 2)			
**DONOR TYPE**					
Living		8 (22.2%)			
Deceased		28 (77.8%)			
**NUMBER OF HLA-A/B/DR-MM**					
0–2		8 (22.2%)			
3–4		16 (44.4%)			
5–6		12 (33.3%)			
CMV serostatus^a^ donor (D)/recipient (R) n (%)		D–/R–	5 (16.7%) (0 infectious episodes)		
		D+/R–	8 (25.0%) (6 primary infections)		
		D–/R+	10 (22.2%) (4 reactivations)		
		D+/R+	13 (36.1%) (3 reactivations)		
**Immunosuppression**	**Initial**	**Month 1**	**Month 3**	**Month 6**	**Month 12**^**b**^
Tacrolimus/cyclosporine A	36/0 (100/0%)	33/1 (91.7/2.8%)	32/2 (88.9/5.6%)	32/3 (88.9/8.3%)	28/4 (77.8/11.1%)
mTOR inhibitor	0 (0%)	2 (5.6%)	2 (5.6%)	1 (2.8%)	3 (8.3%)
Methylprednisolone	36 (100%)	36 (100%)	36 (100%)	36 (100%)	35 (97.2%)
Mycophenolate mofetil	36 (100%)	36 (100%)	36 (100%)	36 (100%)	35 (97.2%)
Basiliximab	35 (97.2%)	0 (0%)	0 (0%)	0 (0%)	0 (0%)
Rituximab	2 (5.6%)^c^	0 (0%)	1 (2.8%)^d^	0 (0%)	0 (0%)
Antithymocyte globulin	1 (2.8%)	0 (0%)	0 (0%)	0 (0%)	0 (0%)
Tacrolimus levels (ng/mL)^e^ Median (IQR)	n.d.	8.5 (7.0–10.8)	7.2 (5.9–8.1)	6.6 (5.6–7.7)	6.2 (4.7–7.0)
Methylprednisolone (mg/d)^e^ Median (IQR)	n.a.	12 (8-12)	4	4	4
**Infections**		**Month 0 to** **≤1**	**Month 1 to** **≤3**	**Month 3 to** **≤6**	**Month 6 to** **≤12**
Infectious episodes^f^		10 in 8 patients (9 viral, 1 bacterial)	21 in 16 patients (17 viral, 4 bacterial)	20 in 18 patients (16 viral, 4 bacterial)	30 in 19 patients (19 viral, 11 bacterial)
Opportunistic infections^g^		9 in 7 patients	16 in 13 patients	16 in 16 patients	17 in 16 patients

a *R+ patients received preemptive therapy, D+/R- received 3 months of valganciclovir prophylaxis*.

b *Two patients had missing ELISA results in the QuantiFERON monitor test at month 12*.

c *AB0-incompatible transplantations*.

d *Antibody-mediated rejection episode*.

e *Refers to actual trough levels/dosage at the time of analysis*.

f *Viral and bacterial infections, no invasive fungal infections occurred*.

g *BKPyV, CMV, EBV, Hepatitis B, HSV, influenza, VZV, Pneumocystis jirovecii; MM, mismatch; mTOR, mammalian target of rapamycin; SD, standard deviation; IQR, interquartile range*.

Finally, we analyzed whether a single test result was able to predict subsequent infectious episodes. At each time point, the IFN-γ level in each transplant-recipient was associated with infectious episodes in the subsequent time interval. Patients had bacterial and viral infections, whereas no episodes of invasive fungal infection occurred (Table 1). As shown in Figure 5C, IFN-γ levels prior to transplantation were significantly higher in patients with infections in the first month. In contrast, IFN-γ levels at later time points tended to be lower in patients with subsequent infection, although this did not reach statistical significance. When infections were stratified for bacterial and viral infections, IFN-γ levels 3 months after transplantation were lower in patients with subsequent bacterial infections (Figure S2). Likewise, IFN-γ levels 6 months after transplantation were significantly lower in patients with subsequent CMV infection episodes, which largely included primary infections after a three month prophylaxis in D+/R- patients. In contrast, IFN-γ levels at earlier time points did not differ in patients with and without infections (Figure S2). Overall, this indicated that IFN-γ levels were unable to predict infection episodes on an individual basis.

## Discussion

Occurrence of adverse events after transplantation such as infections and rejection episodes critically depend on the net state of immunosuppression of the individual patient. Biomarkers to analyze over- or underimmunosuppression in transplant-recipients are not available. In this study, the utility of the QuantiFERON monitor assay was characterized as a tool to quantify the effect of immunosuppressive drugs on innate and adaptive cellular immune function. Of note, cell-reactivity showed diurnal variations that correlated with endogenous cortisol levels in the absence of iatrogenic immunosuppression. In addition, individual immunosuppressive drugs applied *in vitro* had a dose-dependent decreasing effect on cell-reactivity which was further enhanced when drugs were used in combination. In line with previous observations (18), polyclonal immune function showed a larger interindividual variability. While intra-assay variability was very low, inter-assay reactivity varied considerably over time. This not only held true for clinically stable dialysis patients and long-term transplant-recipients but also for immunocompetent controls with no apparent underlying disease or intercurrent infection. Finally, cell-reactivity was lowest in patients with highest levels of immunosuppressive drugs which was shown both in cross-sectional analyses of patient groups with different levels of immunodeficiency as well as in renal transplant-recipients who were analyzed before and during the first year after transplantation. Despite this strong association of cell reactivity with overall immunosuppression in the various groups, the assay seems of limited utility to individualize dosage of immunosuppression or to predict infectious episodes on an individual basis.

The stimulus used in the QuantiFERON monitor assay comprises an anti-CD3 antibody for polyclonal stimulation of T-cells and a TLR7/8 agonist that triggers components of the innate immune response. This allows polyclonal stimulation of blood samples and elicited a strong immune response in CD4 and CD8 T-cells as well as in NK-cells, whereas B-cells remained unresponsive. Among a variety of cytokines tested, IFN-γ was predominantly secreted, and therefore best suited as readout for reactivity of immune cells. Although the overall number of NK-cells was generally lower than that of CD4 or CD8 T-cells (data not shown), the absolute number of IFN-γ secreting cells was comparable for all three lymphocyte subpopulation. Thus, our results show that NK-cells make a similar contribution to IFN-γ secretion in this assay as CD4 and CD8 T-cells. Among NK-cells, IFN-γ is known to be predominantly secreted by the CD56bright subset (19). Apart from a direct induction by the TLR agonist, IFN-γ secretion from NK-cells may also have been indirectly amplified by stimulation-induced T-cell cytokines such as IL-2 (20).

We found that IFN-γ levels were lower in dialysis patients than in controls which may be related to uremic immunodeficiency (21). In transplant recipients, the effects of immunosuppressive drugs present in the plasma of patients *in vivo* or of drugs added *in vitro* can be studied in clinically relevant dosages, as the stimulus is directly added to whole blood. Of note, the assay is very sensitive toward immunosuppressive substances, as even diurnal variations in endogenous cortisol levels inversely correlated with IFN-γ levels in the absence of iatrogenic immunosuppressive drugs, which is important to consider when interpreting test results for use in clinical practice. It would have been informative to study the effect of individual immunosuppressive drugs such as calcineurin inhibitors on cellular immune function by comparing areas under the curve (AUC) for IFN-γ and individual drugs. However, as we did not have patients on drug monotherapy, we chose to perform an *in vitro* stimulation of blood samples in the presence of clinically relevant drug concentrations. This led to a dose-dependent decrease in IFN-γ secretion, and combined action of steroids and calcineurin inhibitors led to a further decrease in IFN-γ production. Similar results were observed using a flow-cytometric assay after 6 h stimulation (7, 17). Moreover, our observations *in vitro* are compatible with results in patients on a routine immunosuppressive drug regimen that included basiliximab, calcineurin inhibitors (largely tacrolimus) and steroids, as IFN-γ secretion was lowest in periods of highest drug levels early after transplantation. This is conceivable for T-cells, as steroids interfere with cytokine induction by suppressing general immune function, and calcineurin inhibitors act by inhibiting the phosphatase activity of calcineurin in T-cells (22). In addition, calcineurin inhibitors were also shown to dose-dependently inhibit IFN-γ secretion from NK-cells (20). More drastic effects on measurable IFN-γ seem to act after T-cell depleting therapy, as ATG treatment was shown to be associated with a further decrease in IFN-γ secretion due to an overall depletion of cytokine-producing cells (12, 23). Despite the clear dose-dependent effect of individual drugs *in vitro*, IFN-γ secretion and tacrolimus trough levels of patients *in vivo* only showed a poor correlation. This is in line with findings from previous studies (7, 12, 13) and emphasizes the fact that the net inhibition of IFN-γ in patient samples is the result of the inhibitory action of several immunosuppressive drugs. Of note, in long-term transplant recipients, IFN-γ secretion levels are similar to patients prior to transplantation which indicates that tapering of immunosuppressive drugs allow restoration of cellular immune function in the long-term.

In general, low counts of lymphocytes including CD4 T-cells or NK-cells have been associated with adverse events after transplantation such as increased infections and rejection episodes (24, 25). Based on this observation, one may argue that the lowest levels in IFN-γ secretion in the early periods after transplantation may be the result of a relative lymphopenia in this time period. As described above, an association between low lymphocyte counts and IFN-γ secretion was indeed observed in patients after T-cell depleting antibody therapy (12). However, in our patient population where only one patient received T-cell depleting antibodies, IFN-γ levels did not directly correlate with lymphocyte counts. In this situation, assessment of antigen-specific cell functionality may better reflect the individual risk than lymphopenia. This was observed for specific T-cells toward pathogens such as CMV or VZV, where a decrease in the functionality of virus-specific T-cells was more strongly associated with impaired pathogen control than a decrease in T-cell or lymphocyte numbers in general (26). Apart from T-cells, this also seems to hold true for NK-cells. Targeting NK-cell function in a bioassay seems interesting in light of the recent findings that impaired NK-cell function was shown to be superior to predict development of NK-cell dependent infections in transplant-recipients as opposed to quantifying NK-cell numbers (25).

A generalized immune function assay is increasingly discussed as potential tool to monitor immunosuppression or to predict infectious complications of any type. Unfortunately, the clinical utility to monitor under- or overimmunosuppression based on a single test result seems rather limited. This also holds true for prediction of infections on an individual basis, which may primarily be related to the different impacts of immunosuppression on the various types of infections and their clinical manifestations, or on multifactorial effects in case of infections with BKPyV. In addition, cellular immune function can vary considerably over time even in individuals without any clinically apparent disease or infection. This not only holds true for dialysis patients and transplant-recipients, but also for immunocompetent controls. These observations may suggest that cellular immune function is critically influenced even by subtle variations in the individual health status, which deserves further study. It is interesting to note that pre-transplant IFN-γ secretion was significantly higher in patients with infections in the first month as compared to patients without infections. This may suggest that this group of patients requires particularly high numbers of functional cells to control infections at steady state. If these patients receive immunosuppression, susceptibility toward infections may be extraordinarily high. In the post-transplant setting, IFN-γ levels 1 month after transplantation had limited ability to predict individual infection events, as cellular immune function was generally low in all patients. In contrast, at later time points, when immunosuppressive drug dosage was already tapered and antimicrobial prophylaxis largely discontinued, sustained low levels of IFN-γ may indicate failure to reconstitute sufficient immunity and may therefore be a better predictor for subsequent infections. Indeed, the difference in IFN-γ levels between patients with and without subsequent bacterial and CMV infections was highest at 3–6 months after transplantation, although the overall number of patients with infections was expectedly low at later time points after transplantation. Similar results were reported in a Canadian study among transplant-recipients (12, 23), where most pronounced associations of IFN-γ levels with subsequent infection episodes were reported at month 6 after transplantation. Interestingly, in liver-transplant-recipients, IFN-γ secretion 1 week after transplantation had the best ability to predict subsequent infection or rejection events (15). However, as with our study, variability in IFN-γ secretion-levels in all groups with and without infections was rather high which decreases the diagnostic value to predict infections on an individual basis. Likewise, other tests to directly or indirectly evaluate the net state of immunosuppression such as the Cylex Immuknow assay or analysis of viral DNA (Torque teno virus or Epstein Barr virus) have shown associations with infectious episodes, but also suffered from limited predictive capacity for use in clinical practice (27–29).

Our study has limitations. First, only one rejection episode occurred, which precluded assessment to predict rejection. Secondly, the sample size of our longitudinal cohort of 36 patients may be too low to reveal clear associations of immune function with infectious episodes and to define thresholds. Nevertheless, even at time points where differences in IFN-γ levels between patients with and without subsequent infections were most pronounced, prediction was poor based on an individual test result.

In conclusion, the QuantiFERON monitor assay elicits a robust IFN-γ response among T-cell subpopulations and NK-cells. As it can be applied directly from whole blood, it is suited to characterize a dose-dependent inhibitory action of individual immunosuppressive drugs or their combinations in clinically relevant concentrations, and may therefore be interesting for basic research on pharmacodynamic activity of immunosuppressive drugs. Although low levels of IFN-γ were associated with increased susceptibility toward infections in large cohorts of patients, this general immune function assay did not prove valuable to predict infections on an individual basis. Therefore, more specific assays relying on pathogen-specific T-cell stimulation may hold better promise as a personalized approach to predict infection episodes (10, 26).

## Data Availability Statement

The raw data supporting the conclusions of this article will be made available by the authors, without undue reservation, to any qualified researcher.

## Ethics Statement

The studies involving human participants were reviewed and approved by Ethikkommission der Ärztekammer des Saarlandes. The patients/participants provided their written informed consent to participate in this study.

## Author Contributions

MS and SM designed the study with support of CA, JM, and US. SM performed all experiments. SM and MS performed analyses and wrote the manuscript. CA, JM, MW, and US recruited patients and provided clinical information. All authors revised the manuscript and approved its content.

## Conflict of Interest

MS has received partial funding of this study and free test reagents by Qiagen. Qiagen had no role in the design, analyses, and interpretation of the results. MS has received research support, travel support and/or honoraria from Qiagen, Astellas, Oxfordimmunotech, Biotest and Novartis outside of the submitted work. US has received travel and research support from Astellas outside of the submitted work. The remaining authors declare that the research was conducted in the absence of any commercial or financial relationships that could be construed as a potential conflict of interest.

## Abbreviations

IFN-γ: interferon-γ
TNF-α: tumor necrosis factor α
IL-2: interleukin 2
CMV: cytomegalovirus
BKPyV: BK polyomavirus
mTOR: mamalian target of rapamycin
MMF: mycophenolate mofetile
TLR: toll like receptor
IQR: interquartile range
SD: standard deviation.
