# Supplementary material for: A Polyclonal Immune Function Assay Allows Dose-Dependent Characterization of Immunosuppressive Drug Effects but Has Limited Clinical Utility for Predicting Infection on an Individual Basis
Source: Front Immunol. 2020 May 15;11:916. doi: 10.3389/fimmu.2020.00916 (PMC7243819; doi:10.3389/fimmu.2020.00916)

Supporting Fig. S1: IFN-γ as the dominant cytokine produced after stimulation. Whole blood samples from 14 healthy controls (see figure 1) were stimulated for 20 h and **(A)** the cytokines IFN-γ, IL-2 and TNF-α were co-stained in CD4 and CD8 T-cells. Cytokine-producing cells were set to 100% and a total of seven subpopulations of cytokine producing cells are displayed. **(B)** NK-cells were analyzed for production of IFN-γ and/or TNF-α positive subpopulations. Only two cytokines could be analyzed due to restrictions in fluorescence channels. **(C)** IFN-γ, IL-4 and IL-17 producing cells among CD4 and CD8 T-cells were analyzed after stimulation with the lyosphere. Statistical analysis was performed using the Friedman test with Dunn´s post-test.

Supporting Fig. S1:

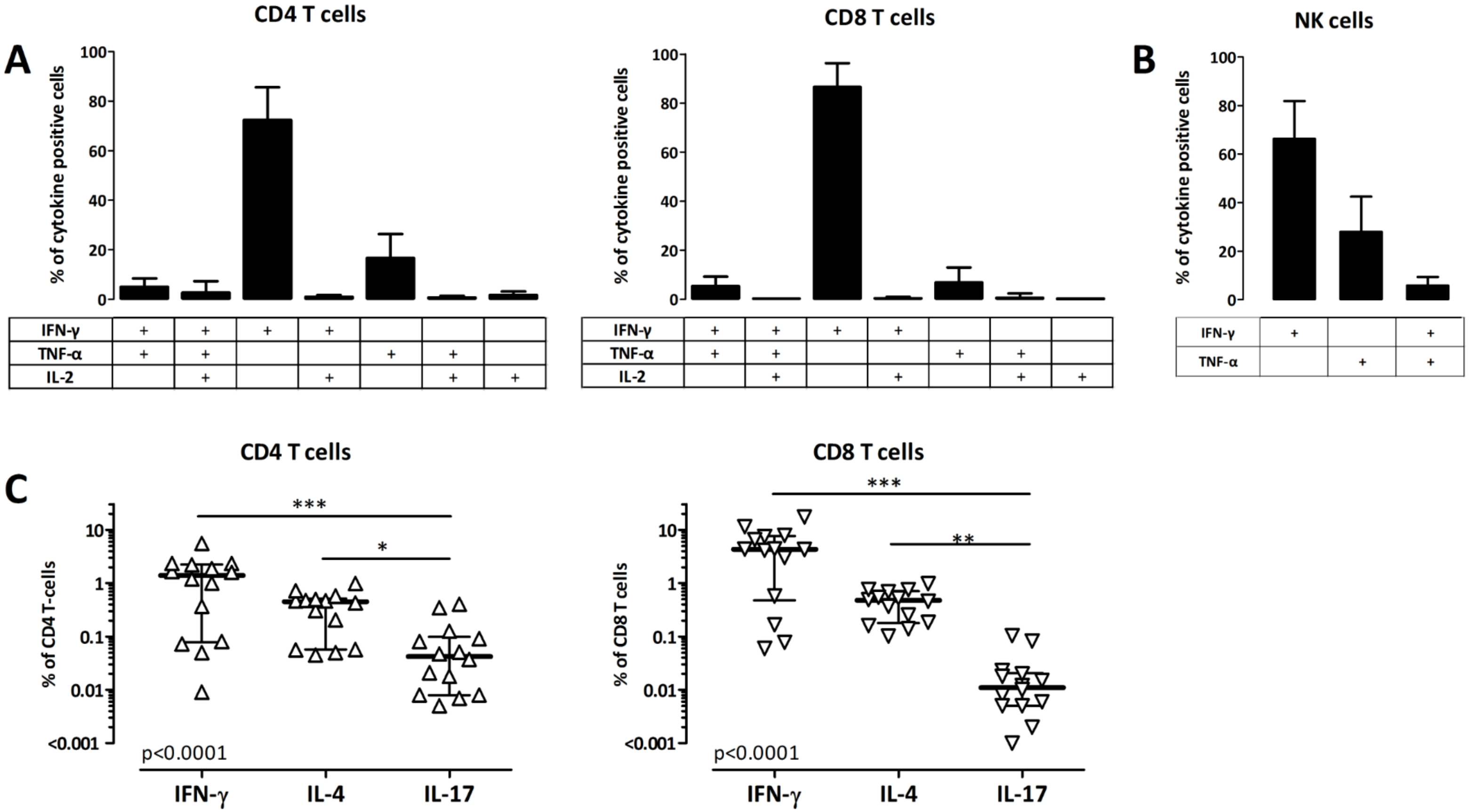

Supplement: Supplementary file 1 [file Image_1.pdf]
