# Supplementary material for: A Polyclonal Immune Function Assay Allows Dose-Dependent Characterization of Immunosuppressive Drug Effects but Has Limited Clinical Utility for Predicting Infection on an Individual Basis
Source: Front Immunol. 2020 May 15;11:916. doi: 10.3389/fimmu.2020.00916 (PMC7243819; doi:10.3389/fimmu.2020.00916)

**Supporting figure S2: Association of IFN-γ secretion levels with bacterial or viral episodes.** IFN-γ secretion-levels of 36 renal transplant-recipients were analyzed before, as well as 1, 3, and 6 months after transplantation (see figure 5B). Episodes of bacterial and viral infections were recorded throughout the first year after transplantation. IFN-γ secretion-levels before as well as 1, 3, and 6 months after transplantation were stratified according to whether or not patients underwent a subsequent episode of bacterial or viral infection. Among viral infections, episodes of cytomegalovirus (CMV) and BK polyomavirus infections (BKPyV) are displayed separately. In the graph on CMV infections, CMV seronegative recipients (R) of CMV seronegative donors (D) were excluded due to low risk (D-/R-, n=5). CMV seropositive patients (D+/R+ and D-/R+) are shown by open symbols (n=23), whereas D+/R- patients are indicated by closed symbols (n=8, black if developing primary infection and gray for patients not developing primary infection). CMV infections were considered if viral load was detectable (>450 copies/mL) regardless of clinical symptoms. BKPyV infections were considered if viral load in urine was $>10^7$ copies/mL and/or if viral load in blood was $>10^4$ copies/mL.

**Supporting Fig. S2:**

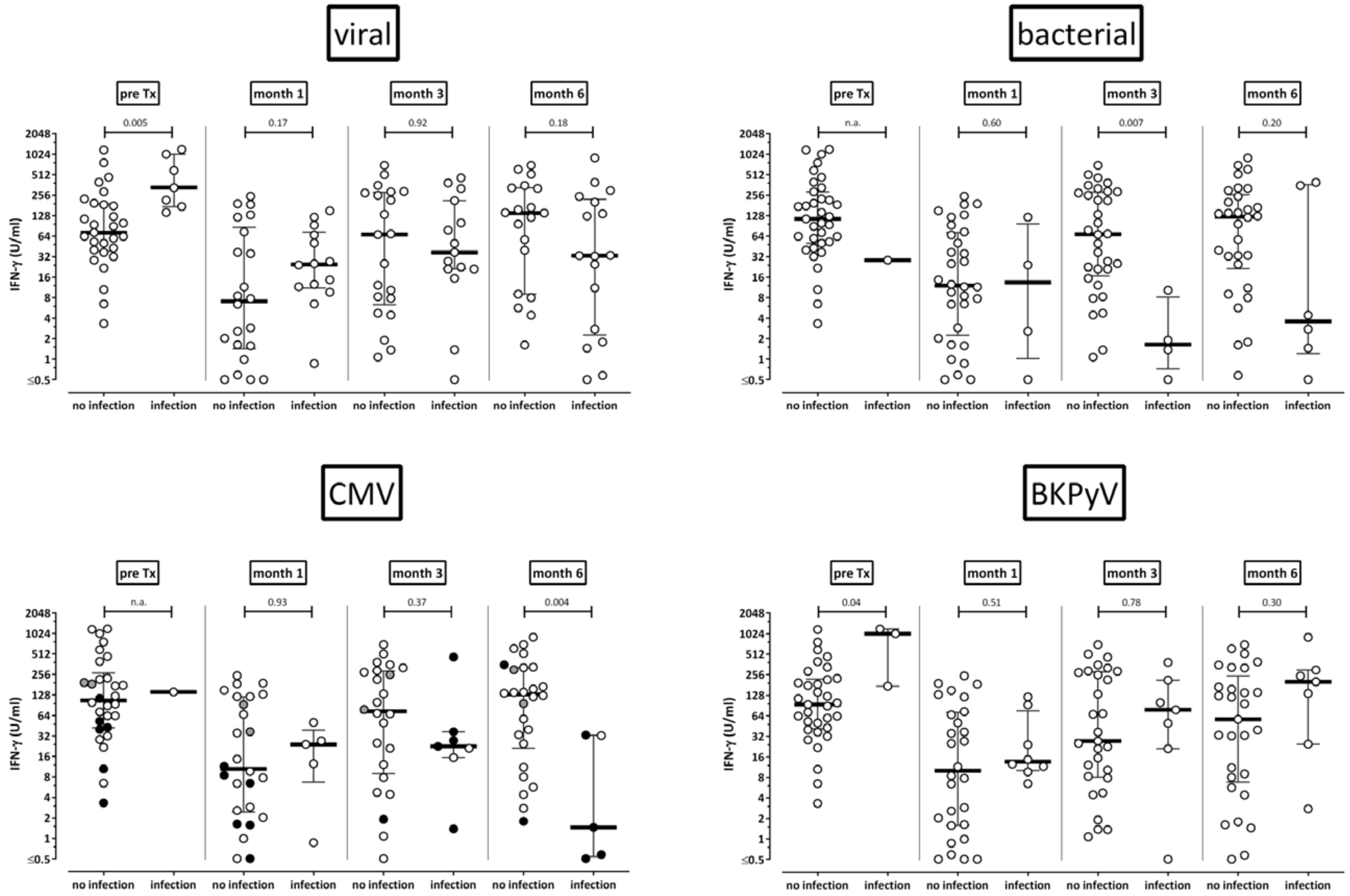

Supplement: Supplementary file 2 [file Image_2.pdf]
