# Supplementary material for: A Polyclonal Immune Function Assay Allows Dose-Dependent Characterization of Immunosuppressive Drug Effects but Has Limited Clinical Utility for Predicting Infection on an Individual Basis
Source: Front Immunol. 2020 May 15;11:916. doi: 10.3389/fimmu.2020.00916 (PMC7243819; doi:10.3389/fimmu.2020.00916)
Supplement: Supplementary file 3 [file Table_1.PDF]

Table S1: Characteristics of controls and patients for cross-sectional analysis-

	Controls	Dialysis patients	Transplant-recipients	
			< 3 months	> 1 year
			after transplantation	
n	51	71	44	61
Years of age (mean±SD)	54.6±19.6	57.9±16.5	53.0±14.5	55.3±14.3
Females n (%)	34 (66.7%)	25 (35.2%)	18 (40.9%)	23 (37.7%)
**Transplant**	n.a.	n.a.		
kidney			44	56
lung			0	5
**Underlying disease**	n.a.			**kidney (n=56)**
Glomerulonephritis		27 (19.2%)	18 (40.9%)	25 (41.0%)
Polycystic kidney disease		8 (11.3%)	5 (11.4%)	6 (9.8%)
Vascular/hypertensive nephropathy		10 (14.1%)	9 (20.5%)	9 (14.8%)
Diabetes mellitus I or II		9 (6.4%)	2 (4.6%)	6 (9.8%)
tubulointerstitial nephritis		5 (7.0%)	5 (11.4%)	3 (4.9%)
unknown/other		12 (16.9%)	5 (11.4%)	7 (11.5%)
				**Lung (n=5)**
COPD				2 (3.3%)
Cystic fibrosis				1 (1.6%)
pulmonary fibrosis				2 (3.2%)
**Renal replacement**	n.a.		n.a.	n.a.
CAPD		4 (5.6%)		
hemodialysis		67 (94.4%)		
Years of renal replacement therapy (mean±SD)		4.8±4.1[1]	5.0±3.9[2]	4.7±3.8[2] (n=56)
Time after transplantation (mean±SD)	n.a.	n.a.	5.1±1.9 weeks	3.3±4.0 years

**Number of previous transplants**	n.a.	n.a.	first (n=42)/<br>second (n=2)	first (n=54)/<br>second (n=7)
**Number of HLA-A/B/DR MM**				kidney n=56
0-2			9 (20.5%)	13 (23.2%)
3-4			21 (47.7%)	23 (41.1%)
5-6			14 (31.8%)	20 (35.7%)
**Immunosuppression**	n.a.		**Initial/<br>time of analysis**	**Initial/<br>time of analysis**
tacrolimus/cyclosporine A			44/0 (100%/0%)/<br>40/2 (90.9%/4.5%))	55/6 (90.2%/9.8%)/<br>43/8 (70.5%/13.1%)
mTOR inhibitor			0 (0%)/<br>2 (4.5%)	0 (0%)/<br>9 (14.8%)
Methylprednisolone/decortin H		1 (1.4%)	44 (100%)/<br>44 (100%)	61 (100%)/<br>56 (95.1%)
mycophenolate mofetil			44 (100%)/<br>44 (100%)	52 (85.2%)/<br>55 (90.2%)
azathioprine			0 (0%)/<br>0 (0%)	9 (14.8%)/<br>4 (6.6%)
Basiliximab			43 (97.7%)/<br>0 (0%)	60 (98.4%)/<br>0(0%)
Rituximab			2 (4.5%)/<br>1 (2.3%)	2 (3.3%)/<br>0 (0%)
Antithymocyte globulin			1 (2.3%)/<br>0 (0%)	1 (1.8%)/<br>0 (0%)
tacrolimus (ng/mL)[3]<br>median (IQR)			8.5 (7.2-10.6)	6.2 (4.7-7.3)

[1]refers to time at analysis; [2]refers to time at transplantation; [3]refers to actual trough levels at the time of analysis; CAPD, continuous peritoneal dialysis; mTOR, mammalian target of rapamycin; SD, standard deviation.
